# Dynamics of serum levels of TNF-α in a longitudinal follow-up study in 98 patients with juvenile idiopathic arthritis treated with anti-TNF-α biological drugs

**DOI:** 10.1007/s10067-024-07012-4

**Published:** 2024-05-22

**Authors:** N. Morozova, M. Zajc Avramovič, G. Markelj, N. Toplak, T. Avčin

**Affiliations:** 1 Department of Allergology, Rheumatology and Clinical Immunology, University Children’s Hospital, University Medical Centre Ljubljana, Bohoričeva Ulica 20, SI-1525, 1000 Ljubljana, Slovenia; 2 Department of Pediatrics, Faculty of Medicine, University of Ljubljana, Ljubljana, Slovenia

**Keywords:** Adalimumab, Cytokines, Etanercept, Juvenile idiopathic arthritis, Tumour necrosis factor-alpha

## Abstract

**Objective:**

To determine the dynamics of serum levels of TNF-α in patients with juvenile idiopathic arthritis (JIA) treated with anti-TNF-α biological drugs and investigate their association with the disease activity.

**Methods:**

We conducted a single-centre, observational cohort study in 98 patients with JIA (30 boys, 68 girls, mean age 11.3 years) treated with anti-TNF-α biological drugs. Clinical examinations and laboratory assessments of serum levels of TNF-α were performed before starting therapy with biological drug and at 6-month intervals afterwards up to 2.5 years.

**Results:**

The analysis of serum levels of TNF-α in relation to the disease activity states showed the highest mean serum levels of TNF-α in patients on etanercept who had low disease activity states and in patients on adalimumab who had inactive disease. The correlation analysis in patients with JIA treated with etanercept or adalimumab showed a weak negative correlation between the serum levels of TNF-α and JADAS10 scores (*p* = 0.007), (*r* =  − 0.177).

**Conclusion:**

The assessment of serum levels of TNF-α in children with JIA during treatment with etanercept or adalimumab is not a reliable biomarker of disease activity or immunological remission. Longitudinal measurement of TNF-α has no added clinical value in patients with JIA treated with anti-TNF-α biological drugs.

**Table:** 

**Key Points** *• There is limited evidence regarding the effect of anti-TNF therapy on serum concentrations of TNF-α in patients with juvenile idiopathic arthritis* *• Our study showed an increase in the serum level of TNF-α after the initiation of therapy with either etanercept or adalimumab, which was more significant in patients with inactive or low disease activity* *• Serum TNF-α is most likely not biologically active during therapy with TNF-α inhibitors and therefore not a reliable biomarker of disease activity or immunological remission in patients with juvenile idiopathic arthritis*

## Introduction

Juvenile idiopathic arthritis (JIA) is the most common rheumatic disease in the paediatric population, characterised by chronic inflammation in one or more joints for a minimum of 6 weeks. A significant role in developing and maintaining a persistent inflammatory response and in the process of joint destruction in JIA is attributed to cytokines [1]. A key cytokine involved in the pathogenesis of JIA is tumour necrosis factor-alpha (TNF-α), which can stimulate the expression of other proinflammatory cytokines, including interleukins IL-1β, IL-6, and IL-8, leading to a protracted inflammatory response [2, 3]. In the normal physiological state, proinflammatory cytokines, including TNF-α, are sustained in a state of balance with anti-inflammatory cytokines, such as IL-10.

Inflammation is associated with the accumulation of inflammatory cells, mostly type 1 helper T cells (Th1) and macrophages, together with B cells, plasma cells, and dendritic cells (DCs). In JIA, TNF-α secreted from Th1 cells and macrophages activates synovial fibroblasts, encourages epidermal hyperplasia, and engages inflammatory cells. After the process of activation by diverse cytokines, synovial fibroblasts overexpress cathepsins and matrix metalloproteinases (MMPs), followed by collagen and proteoglycan breakdown [4, 5]. Consequently, cartilage and bone are destroyed; eventually, joint erosion may occur. Osteoclasts are equally significant for the development of JIA pathology throughout TNF-α activation and the promotion of synovial hyperplasia and angiogenesis.

Several studies revealed increased levels of TNF-α, IL-6, and IL-8 in patients with various JIA subtypes. These studies investigated levels of serum TNF-α, IL-1β, IL-6, and IL-17 in JIA patients with active disease compared to those with inactive disease and healthy controls [1, 6–8]. In contrast, there is very limited information concerning the influence of biological drugs on the serum concentrations of TNF-α during treatment.

Our study aimed to determine the dynamics of serum levels of TNF-α in patients with JIA treated with biological drugs at different time points during treatment. Moreover, we investigated the association between the serum cytokine levels and disease activity markers during biological therapy to assess the diagnostic and prognostic value of serum levels of TNF-α as an immunological remission biomarker in patients with JIA treated with biologics.

## Patients and methods

### Study design

We conducted a single-centre, bidirectional, observational cohort study in patients with JIA treated with biological drugs between July 2018 and January 2021 at the University Children's Hospital Ljubljana. The data were collected retrospectively for patients who started biological therapy before July 2018 and prospectively for patients who newly initiated biological therapy after July 2018. The inclusion criteria were a definite diagnosis of JIA according to the International League of Associations for Rheumatology criteria [9] and previous or current treatment with TNF-α inhibitor or IL-6 inhibitor. The study was approved by the National Medical Ethics Committee of the Republic of Slovenia. All participants gave their informed consent prior to their inclusion in the study. Informed consent was provided by legal guardians for all minors under the age of 18 years.

### Study population

The study involved a group of 98 patients with JIA (30 boys, 68 girls) who were longitudinally followed at the University Children’s Hospital Ljubljana, Slovenia. The mean age of participants at the time of inclusion was 11.3 years (ranging from 1.3 to 21.2 years), and the mean disease duration was 7.2 years (ranging from 1.2 to 18.5 years).

Classification of JIA was made at least 6 months after the onset of the disease. Eighteen patients had rheumatoid factor (RF) negative polyarthritis, 8 RF positive polyarthritis, 37 persistent oligoarthritis, 19 extended oligoarthritis, 9 enthesitis-related arthritis, and 7 psoriatic arthritis. All patient data were prospectively entered into the hospital clinical database (Think!Med Clinical™, Better, Slovenia). Sixty-seven patients were already taking biological drugs before the study initiation, and 31 patients started taking biological therapy during the study and were prospectively enrolled.

Standardised clinical assessment and laboratory data were prospectively collected, including medical history, general and musculoskeletal status, laboratory markers of disease activity (C-reactive protein (CRP), erythrocyte sedimentation rate (ESR), and serum concentration of TNF-α).

JIA disease activity score was calculated at each study visit. Disease activity categories were defined based on the previously published JADAS10 cut-offs for patients with oligoarthritis or polyarthritis [10]. All patients enrolled in the study had active disease at baseline before starting therapy with a biological drug (JADAS10 ≥ 1.1). During the study, 54/98 (55.1%) patients achieved clinical remission on medication and 3/98 (3.06%) patients achieved clinical remission off medication [11].

### The measurement of serum TNF-α

Blood was collected to determine cytokine concentrations during routine venipuncture performed in the hospital or outpatient clinic for periodic assessment of laboratory tests. Initial serum TNF-α levels were measured before treatment with a biological drug (baseline). After the start of biological therapy, the 1st follow-up measurement was performed 3–6 months after the beginning of therapy, the 2nd follow-up measurement after 6–12 months, the 3rd follow-up measurement after 1–1.5 years, and the 4th follow-up measurement after 1.5–2.5 years. Patients in the retrospective group had additional measurements at various longer time intervals up to 10 years after the start of the biological therapy.

The concentration of TNF-α in serum was determined using the TNF-α Human ELISA Kit (Invitrogen, USA) according to the manufacturer's instructions. The analyses were conducted at the Institute of Microbiology and Immunology, Faculty of Medicine, University Ljubljana.

### Statistical analyses

All variables investigated in the study were not normally distributed according to the Kolmogorov–Smirnov and Shapiro–Wilk tests; nonparametric tests were used for data analysis. A Mann–Whitney U test was used to discover the difference between two conditions, a Kruskal–Wallis test to discover the difference between more than two groups, and Spearman's correlation coefficient was used to discover associations between numeric variables. Statistical analyses were performed using IBM SPSS Statistics software.

## Results

### Patient characteristics

Based on the current treatment approaches, our patient cohort was grouped according to the clinical phenotypes of JIA rather than specific classification criteria [12–14]. Of 98 patients, 43 (43.9%) had oligoarticular, and 55 (56.1%) polyarticular disease. In the polyarticular disease group, 19 patients had extended oligoarthritis, 18 RF-negative polyarthritis, 8 RF-positive polyarthritis, 7 psoriatic arthritis, and 3 enthesitis-related arthritis. In the oligoarticular disease group, 37 patients had persistent oligoarthritis and 6 enthesitis-related arthritis.

During the study, 60 patients were receiving etanercept and 52 adalimumab. Eighty-two patients had previously received methotrexate, 9 sulphasalazine, 4 leflunomide, and 3 mycophenolate mofetil (Table 1).

**Table 1:** Treatment characteristics of 98 patients with juvenile idiopathic arthritis included in the study

		Oligoarticular JIA	Polyarticular JIA	Total
Number of patients (% of all patients)		43 (43.9%)	55 (56.1%)	98
Total number of patients treated with biological drug	Adalimumab	20 (38.5%)	32 (61.5%)	52
	Etanercept	25 (41.7%)	35 (58.3%)	60

Due to the ineffectiveness of the therapy, we switched biological therapy in 3 patients with oligoarticular disease from etanercept to adalimumab and in 9 patients with polyarticular disease as follows: 6 patients from etanercept to adalimumab, and in 3 patients from adalimumab to etanercept. For the retrospectively included patients, the average duration of biological DMARD intake prior to the study entry was 3.2 years (ranging from 3 months to 9 years).

### The level of TNF-α before and during the therapy with etanercept and adalimumab

The initial mean level of TNF-α at first measurement before therapy with etanercept was 34.8 ± 34.9 pg/ml and in patients before therapy with adalimumab 32.6 ± 31.7 pg/ml, respectively (Table 2). The baseline level of TNF-α (before therapy with etanercept) was significantly lower than all subsequent determinations of TNF-α during the therapy with etanercept for 1st, 2nd, 3rd (*p* < 0.0001), and 4th follow-up measurement (*p* = 0.001) (Table 2). Similarly, the baseline level of TNF-α (before therapy with adalimumab) was significantly lower than the level of TNF-α at the 1st (*p* = 0.006) and 2nd follow-up measurements (*p* = 0.002). There were no significant differences between the baseline level of TNF-α and the 3rd and 4th follow-up measurements (Table 2).

**Table 2:** Mean cytokine levels at baseline and follow-up measurements

Cytokines (*n* = 98)	Baseline value	1st F/u	2nd F/u	3rd F/u	4th F/u	*p*
Follow-up levels of TNF-α on therapy with etanercept	(*n* = 49) 34.78 (± 34.85)	(*n* = 49) 91.04 (± 52.06)	(*n* = 35) 94.70 (± 85.76)	(*n* = 21) 75.06 (± 46.54)	(*n* = 13) 77.95 (± 56.89)	< 0.0001
Follow-up levels of TNF-α on therapy with adalimumab	(*n* = 36) 32.59 (± 31.66)	(*n* = 36) 48.37 (± 33.91)	(*n* = 23) 51.91 (± 29.13)	(*n* = 13) 41.56 (± 26.38)	(*n* = 7) 47.90 (± 32.53)	0.014

± standard deviation

### The serum level of TNF-α in relation to the disease activity states in patients treated with etanercept and adalimumab

**Fig. 1:**
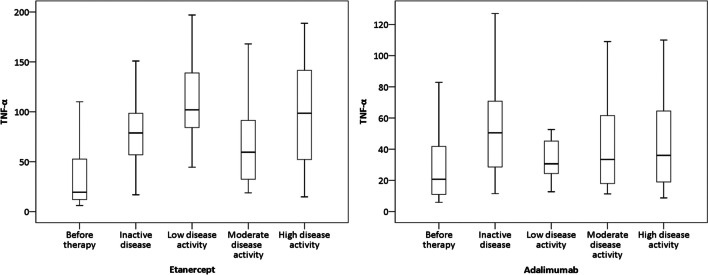
The serum levels of TNF-α before and during the therapy with etanercept and adalimumab in patients with JIA stratified according to the disease activity status

The analysis of serum levels of TNF-α in relation to the disease activity states (inactive disease, low, moderate, and high disease activity) in patients treated with etanercept or adalimumab is presented in Fig. 1. The mean level of TNF-α was significantly higher in the group of patients with inactive disease (*p* < 0.001), low disease activity (*p* < 0.001), moderate disease activity (*p* < 0.001), or high disease activity (*p* < 0.001) states, compared to patients before the therapy with etanercept. The highest mean serum level of TNF-α in patients treated with etanercept was observed in patients with low disease activity. There was a significant difference in the level of TNF-α between inactive disease and low disease activity (*p* = 0.040) and between low and moderate disease activity (*p* = 0.022). Between other groups, there were no significant differences in the levels of TNF-α for patients on therapy with etanercept.

The serum level of TNF-α was significantly higher in the group of patients treated with adalimumab with inactive disease (*p* < 0.001) and moderate disease activity (*p* = 0.049) compared to patients before the therapy with adalimumab. Between other groups, there were no significant differences in the serum levels of TNF-α. The highest mean serum level of TNF-α in patients treated with adalimumab was observed in patients with inactive disease states.

### Dynamics of JADAS10 scores and serum levels of TNF-α during the study period in patients with oligoarticular and polyarticular *JIA* treated with anti-TNF-α therapy

**Fig. 2:**
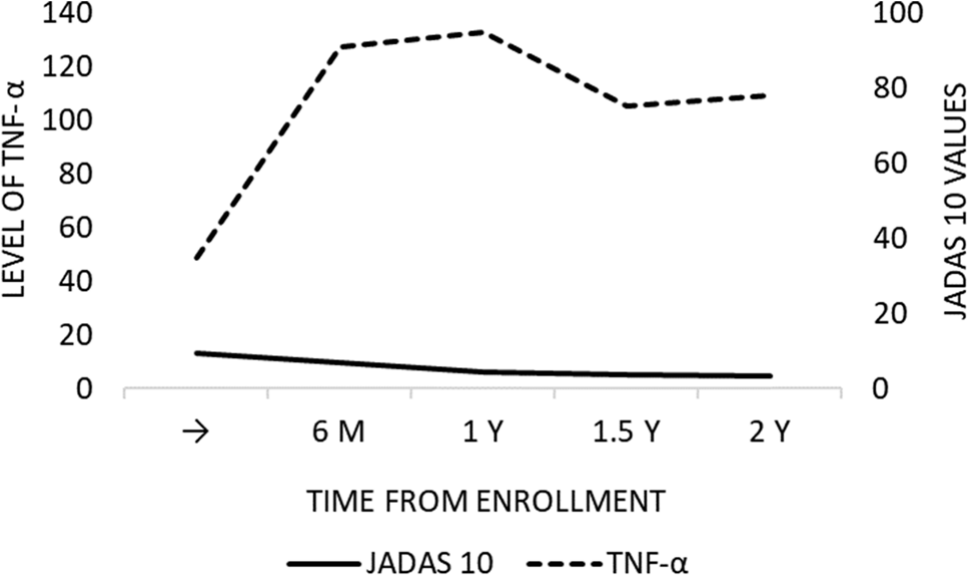
Mean serum levels of TNF-α and mean values of JADAS10 scores during the time from enrolment in patients with oligoarticular and polyarticular JIA

Eighty-six percent of patients with oligoarticular and polyarticular JIA had increased serum levels of TNF-α, and only 13.8% of cases had serum levels of TNF-α within the normal range. Comparison of the dynamics of JADAS10 and levels of TNF-α over time showed that the levels of TNF-α progressively increased after the initiation of anti-TNF-α therapy, while the JADAS10 scores were slowly decreasing from the first to the last time point during the follow-up as presented in Fig. 2.

### Correlation between JADAS10 scores and the serum levels of TNF-α

**Fig. 3:**
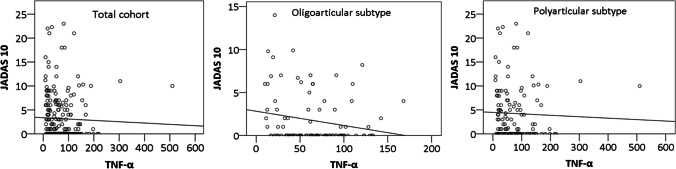
The correlation between the JADAS10 scores and serum levels of TNF-α in patients with JIA on therapy with etanercept and adalimumab

The analysis of the association between the serum levels of TNF-α and the activity of the disease (JADAS10 scores) in patients on therapy with adalimumab or etanercept in the total cohort of patients with JIA showed a weak negative correlation (Fig. 3) between the level of TNF-α and JADAS10 scores (*p* = 0.007), (*r* =  − 0.177). Similarly, a statistically significant negative correlation was found between the serum levels of TNF-α and JADAS10 scores during therapy with adalimumab or etanercept (*p* = 0.040) in a subgroup of patients with oligoarticular JIA (*r* =  − 0.207), but there was no statistically significant correlation between the serum levels of TNF-α and JADAS10 scores in patients with polyarticular JIA (*r* =  − 0.171).

## Discussion

Our study aimed to assess the dynamics of serum levels of TNF-α in relation to the disease activity in patients with JIA treated with biological drugs. We also wanted to evaluate the diagnostic and prognostic values of TNF-α as an immunological remission marker in JIA.

A prominent finding of our study was a significant increase in the serum levels of TNF-α after initiation of treatment with anti-TNF-α therapy either with etanercept or adalimumab. The serum levels of TNF-α during therapy with etanercept were higher than during therapy with adalimumab. The highest levels of TNF-α (mean concentration 123.52 pg/ml) in patients on etanercept were recorded in those who had low disease activity.

An increase in the serum level of TNF-α after initiation of treatment with etanercept was previously described in adult patients with TNF receptor-associated periodic syndrome, multiple myeloma, orthoclone (OKT)-associated syndrome, and metastatic breast cancer [15–18]. An increase in the serum levels of TNF-α was also observed in adult patients with ankylosing spondylitis (AS) and rheumatoid arthritis (RA) treated with etanercept despite clinical improvement [19].

Increased levels of TNF-α in patients treated with etanercept compared to other JIA patients and the control group were also observed in paediatric research. One study found the highest TNF-α levels in those JIA patients who were in clinical remission clinical remission; however, the measurements were only taken cross-sectionally in patients already on therapy with etanercept and not before therapy initiation [20]. Another study found that large increases in TNF-α measured 6 weeks after the initiation compared to 6 weeks before the initiation of etanercept therapy suggested lower levels of etanercept treatment discontinuation [21].

A few theories were proposed to explain the increase in TNF-α levels during etanercept therapy. Etanercept is a TNF-α Fc-fusion protein that can bind and neutralise TNF-α, similar to the naturally occurring soluble TNF-α receptor, but can also stabilise TNF-α and increase its serum half-life. Thus, patients receiving etanercept therapy may have higher concentrations of TNF-α, but this TNF-α may not be biologically active.

A similar increase in the serum levels of TNF-α was reported in adult patients with RA receiving adalimumab [22]. The study demonstrated that the levels of TNF-α stabilised after an initial increase and remained stable for 2 years of follow-up, and did not decrease in patients with RA in remission. In addition, TNF-α levels also increased after the administration of adalimumab biosimilar to healthy volunteers, reaching values comparable to those in patients with RA. The data from this study showed that the complex formation between TNF-α and adalimumab extends TNF-α half-life. These data indicate that serum levels of TNF-α during adalimumab therapy do not reflect inflammatory activity in the joints and that most TNF-α is probably not caused by pathological processes. However, no significant increase in TNF-α levels was observed in a study of JIA patients during four follow-ups on adalimumab therapy compared to baseline measurement before therapy initiation [23]. The authors suggest that adalimumab may disguise TNF-α epitopes, which are usually detected by laboratory assays.

The quantification of TNF-α presents a major challenge for several reasons. TNF-α is rapidly eliminated from the blood flow as its half-life lasts only several minutes [24]. Therefore, the concentrations of TNF-α in the blood flow are low, close to the detection threshold in most immunological tests, even in the case of an active disease [25]. TNF-α is also easily decomposable in biological samples due to the constant exchange of monomeric subunits. Lastly, the trimeric structure of TNF-α can be easily disrupted, for instance, when serum samples are frozen or thawed, which directly affects TNF-α quantification [26]. Therefore, it is unlikely that pretreatment levels of TNF-α are reliable biomarkers of disease activity. This is also in line with our findings since patients with JIA with high disease activity before initiation of treatment with biologics did not have high serum levels of TNF-α.

The analysis of serum levels of TNF-α in relation to the disease activity states showed the highest mean serum levels of TNF-α in patients on etanercept (123.52 pg/ml) who had low disease activity state and in patients on adalimumab (55.35 pg/ml) who had inactive disease.

During treatment with TNF-α inhibitors, the TNF-α bound to a TNF-α inhibitor has an extended half-life since TNF-α inhibiting antibodies themselves have a very long half-life of several weeks [23]. This may also explain the observed increase in the serum levels of TNF-α in our study after initiation of treatment with anti-TNF-α biological drugs. Most likely, TNF-α is not biologically active during therapy with TNF-α inhibitors and cannot be used as a reliable biomarker for immunological remission in JIA.

Based on the number of affected joints, we divided patients from our study into two groups (oligoarticular and polyarticular JIA) and assessed the impact of the disease subtype on the serum level of TNF-α. The study demonstrated that the disease subtype did not affect the correlation between the serum levels of TNF-α and disease activity (JADAS10 scores). The mean levels of TNF-α gradually increased both in patients with oligoarticular and polyarticular disease subtypes, which was associated with a gradual decrease in disease activity (JADAS10 scores). The highest serum levels of TNF-α were recorded 2.5 years after the initiation of therapy with TNF-α inhibitor when most of our patients had low disease activity or inactive disease.

One of the drawbacks of our study is that the follow-up clinical assessments and laboratory measurements were performed according to the schedule of patient visits in the outpatient clinic and were not entirely uniform for all patients. The time frame for individual follow-up measurements could span from 3 to 6 months and may have influenced the serum levels of TNF-α during the study period.

In summary, based on the results of our study, we presume that the assessment of serum levels of TNF-α in children with JIA during treatment with TNF-α inhibitors (etanercept or adalimumab) is not a reliable biomarker of disease activity or immunological remission. The highest mean serum levels of TNF-α were observed in patients with low or inactive disease states, and the correlation analysis revealed a weak negative correlation between the serum levels of TNF-α and disease activity JADAS10 scores. It appears that longitudinal measurement of TNF-α has no added clinical value in patients with JIA treated with anti-TNF-α biological drugs.

## Data Availability

The data that support the findings of this study are available on request from the authors.
